# Therapeutic agents for Alzheimer’s disease: a critical appraisal

**DOI:** 10.3389/fnagi.2024.1484615

**Published:** 2024-12-09

**Authors:** Marta Weinstock

**Affiliations:** Institute for Drug Research, School of Pharmacy, Faculty of Medicine, The Hebrew University, Jerusalem, Israel

**Keywords:** antibodies against beta amyloid, ***β*** and ***γ*** secretase inhibitors, cholinesterase inhibitors, ladostigil, rosiglitazone, TNFα antagonists

## Abstract

Alzheimer’s disease (AD) is the most common form of dementia. Mutations in genes and precursors of *β* amyloid (Aβ) are found in the familial form of the disease. This led to the evaluation of seven monoclonal antibodies against Aβ in subjects with AD, two of which were approved for use by the FDA. They caused only a small improvement in cognitive function, probably because they were given to those with much more prevalent sporadic forms of dementia. They also have potentially serious adverse effects. Oxidative stress and elevated pro-inflammatory cytokines are present in all subjects with AD and are well correlated with the degree of memory impairment. Drugs that affect these processes include TNFα blocking antibodies and MAPK p38 inhibitors that reduce cognitive impairment when given for other inflammatory conditions. However, their adverse effects and inability to penetrate the brain preclude their use for dementia. Rosiglitazone is used to treat diabetes, a risk factor for AD, but failed in a clinical trial because it was given to subjects that already had dementia. Ladostigil reduces oxidative stress and suppresses the release of pro-inflammatory cytokines from activated microglia without blocking their effects. Chronic oral administration to aging rats prevented the decline in memory and suppressed overexpression of genes adversely affecting synaptic function in relevant brain regions. In a phase 2 trial, ladostigil reduced the decline in short-term memory and in whole brain and hippocampal volumes in human subjects with mild cognitive impairment and had no more adverse effects than placebo.

## Introduction

Dementia is one of the leading causes of morbidity and mortality worldwide that exerts an immense, negative effect on the quality of life of the subjects, their families and on health care systems. Globally, the total number of people with this condition is expected to reach almost 153 million in 2050 (Li et al., 2022). Alzheimer’s disease (AD), the most common cause of dementia (Hyman et al., 2012), is a progressive neurodegenerative disorder, characterized by the degeneration of cholinergic neurons in the nucleus basalis, and the presence of extracellular plaques beta amyloid (Aβ) and intracellular neurofibrillary tangles composed of phosphorylated tau. AD presents with an impairment in early episodic memory, followed by a gradual and progressive deterioration in cognition and behavior. The characteristic features of the familial form (FAD) were originally described by Alois Alzheimer in 1906. It has an age of onset between 30 and 60 years and is associated with autosomal dominant inheritance of mutations in the amyloid precursor protein, presenilin 1 (PSEN1) or PSEN2 genes (Jagust, 2018). Either PSEN1 or PSEN2 can be the catalytic subunit of *γ*-secretase, that generates Aβ from amyloid precursor protein. Aβ has been implicated in synaptic dysfunction, disruption of neural connectivity and neuronal death in a brain region-specific manner (Lacalle-Aurioles and Iturria-Medina, 2023).

In FAD, Aβ-containing plaques appear at least 20 years before any signs of memory impairment (Bateman et al., 2012). While prevention of Aβ formation could provide a treatment option for FAD if started early enough, it represents only about 1% of subjects with AD (Hippius and Neundorfer, 2003). The rest have the sporadic form of AD (SAD), with an age of onset of more than 65 years. Their brains also have Aβ-containing plaques, but so do those of healthy, older people with no overt signs of dementia (Rodrigue et al., 2012). Since no correlation was found between the number of Aβ plaques and the degree of cognitive impairment in individuals with SAD (Bennett et al., 2012), the original hypothesis was changed and soluble oligomers of Aβ proposed as the cause of neurodegeneration (Shankar et al., 2008).

## Mild cognitive impairment

Petersen originally described mild cognitive impairment in Petersen (2004) and applies to the earliest symptomatic stage of cognitive impairment in which either a single or several cognitive domains are compromised to a mild extent, but functional ability is preserved. However, no differences were found in MCI in subjects with or without evidence of Aβ after applying several different psychological different tests (Insel et al., 2018).

## Acetylcholinesterase inhibitors

Acetylcholinesterase (AChE)-positive neurons project diffusely to the cortex from the cholinergic nucleus basalis magnocellularis of Meynert, modulating cortical processing and responses to new and relevant stimuli. The findings that in AD there is a correlation between loss of neurons projecting from the nucleus basalis and the decline in mental status (Whitehouse et al., 1982) led to introduction of AChE inhibitors to preserve acetylcholine levels. Tacrine was the first AChE inhibitor used for the treatment of AD but was withdrawn because of hepatotoxicity (Watkins et al., 1994). Of several others, the two AChE inhibitors most frequently prescribed are donepezil and rivastigmine.

Donepezil, is a selective AChE inhibitor approved in 1996 for mild to moderate AD when administered at a daily dose of 5 mg during the first month of treatment and increased up to 10 mg. The result of a meta-analysis of 5 studies in a total of 1,130 participants showed that 10 mg was associated with a better outcome for cognitive function than placebo after 26 weeks, Alzheimer’s Disease Assessment Scale-Cognitive (ADAS-Cog, range 0 to 70) was −2.67 points. The incidence of adverse effects, insomnia, diarrhea, vomiting, anorexia, and muscle cramps was higher at 12 weeks, but declined by 26 weeks (Birks and Harvey, 2018).

Rivastigmine is a slowly reversible inhibitor of AChE and Butyrylcholinesterase (BuChE). BuChE is distributed in neurons, glia and endothelial cells and BuChE-positive neurons that project to the frontal cortex may play a role in attention and executive function (Bullock and Lane, 2007). While AChE declines in AD, with a loss of cholinergic neurons, BuChE activity progressively increases as the severity of dementia advances. Rivastigmine interacts preferentially with the G1 form of the enzyme found in high levels in the brains of patients with AD. At doses of 6 to 12 mg/day, rivastigmine produced a significant dose related improvement of ≥4-points in the ADAS-cog subscale at 26 weeks after treatment compared to placebo. Adverse effects were nausea and vomiting that were reduced by dose titration (Spencer and Noble, 1998). Rivastigmine is metabolized by its target enzyme and not by CRP450 in the liver. Thus, it can safely be given with the variety of medications used to treat elderly subjects. Since rivastigmine causes less inhibition of AChE in the striatum than in the cortex and hippocampus, it can also be administered to patients with dementia and Parkinson’s disease. None of the AChE inhibitors affect the underlying causes of cholinergic neuron deterioration, thus their effect is only seen as long as acetylcholine is still being released.

## N-methyl-d-aspartate receptor antagonists: memantine

Glutamate is the major excitatory neurotransmitter in the brain that acts on several synaptic receptors including N-methyl-d-aspartate (NMDAR). This receptor plays a fundamental role in synaptic plasticity, and the underlying molecular mechanisms of learning and memory. Activation of NMDARs is also important for the survival of neurons apoptosis (Collingridge and Singer, 1990). However, activation of extra-synaptic NMDARs by the spillover of glutamate from astrocytes or presynaptic terminals results in prolonged Ca2+ influx into the postsynaptic neuron, loss of synaptic function and neuronal cell death. This correlates with the decline in cognition and the development of dementia (Hardingham and Bading, 2010).

The observation that memantine was able to suppress signaling through these extra-synaptic receptors resulted in its approval by the FDA for the treatment of AD. The results of a large number of placebo controlled clinical trials of memantine, summarized in two mega analyses indicate that memantine improves cognitive function, AD-associated behavioral disturbances and activities of daily living in subjects with moderate to severe dementia. However, these effect sizes were small (SMD = −0.09 to −0.27) but adverse effects were mild (Matsunaga et al., 2015). There was no clinically significant benefit from the addition of AChE inhibitors like donepezil (McShane et al., 2019).

## Secretase inhibitors

During the last decade, the pharmaceutical industry has concentrated its efforts to affect the processes leading to neurodegeneration by developing drugs to decrease Aβ. *γ*-secretase is a multi-subunit protease that was identified as responsible for the generation of Aβ, and thus considered a prime therapeutic target in AD (Shoji et al., 1992). This led to the development of *γ*-secretase inhibitors like semagacestat to inhibit the formation of Aβ. However, a phase 3 trial in patients with mild to moderate AD was prematurely stopped because the drug actually worsened several measures of cognitive function (Doody et al., 2013). Like other *γ*-secretase inhibitors, avagacestat and tarenflurbil (Penninkilampi et al., 2016), semagacestat caused serious adverse effects, including cancer, skin related disorders, hypersensitivity reactions, increase in infections and renal failure (Henley et al., 2014).

*β*-secretase inhibitors also prevent formation of Aβ from amyloid precursor protein and their adverse effects are less serious than those of γ-secretase inhibitors. However, verubecestat, atabaques and lanabecestat (all worsened cognitive function in subjects with mild–moderate AD) (Egan et al., 2018; Novak et al., 2020; Wessels et al., 2020). Verubecestat also increased the rate of decline of hippocampal volume, compared to placebo.

## Antibodies against Aβ

Seven injectable, monoclonal antibodies (Abs) have been prepared to remove Aβ fibrils or plaques (Alves et al., 2023). Positron emission tomography scans for Aβ confirmed its removal by the Abs but the effect on memory decline in patients with mild to moderate degrees of AD of aducanumab, solenazumab and bapineuzumab, which bind to Aβ plaques, was barely different from that of placebo (Mullard, 2019; Mahase, 2021; Mahase, 2022). Lecanemab binds to soluble Aβ protofibrils while donanemab binds to insoluble, modified, N-terminal truncated form of β-amyloid present only in amyloid plaques. They were approved by the FDA after two large placebo-controlled trials in more than 800 subjects. The Clinical Dementia Rating Sum of Boxes is a continuous measure of dementia severity and ranges from 0 to 18 and is suitable for the earliest stages of AD. The difference in this measure between lecanemab and placebo, the primary end point in the trial, was only 0.45 points (van Dyck et al., 2023) and for donanemab, 0.7 points (Sims et al., 2023).

Like all the Abs developed so far, lecanemab and donanemab cause various degrees of magnetic resonance imaging-detectable amyloid-related imaging abnormalities, such as cerebral edema or hemorrhage in addition to ventricular enlargement ranging from 23 to 57% (Alves et al., 2023). Recently, the FDA has approved injection once a month of donanemab after a new trial but the company that who developed it warned of potential adverse in some subjects that include headache, dizziness, nausea, difficulty walking, confusion, vision changes and seizures.

Regulatory agencies other than the FDA have conceded that any cognitive improvement by these agents is well below a minimally, clinically important effect (Sperling et al., 2023). Cerebral bleeding proscribes their use in patients on anti-coagulants and those with the ApoE4 gene, a strong risk factor for AD (Mahley, 2016). Furthermore, their high cost, coupled with the need for administration by intravenous injection, once or twice a month, make them unavailable to most subjects with AD. More consideration should be given to other causes of neurodegeneration in order to develop safer, cheaper treatments to prevent the development of AD, by giving them orally to subjects with MCI, the earliest prodromal stage of AD, mild cognitive impairment.

## Other causes of neurodegeneration

Neuronal damage leading to cognitive impairment can be produced by a combination of environmental and genetic factors. These include a high body mass index, diabetes, insulin resistance, hypertension and dyslipidemia disorders (Verdile et al., 2015). Cognitive impairment can occur in diabetic subjects with chronic inflammation through disruption of the blood brain barrier and entry of macrophages (Varatharaj and Galea, 2017). The APOE gene encodes a protein involved in lipid and cholesterol binding and transport. The APOE 4 allele is an important risk factor for SAD (Strittmatter et al., 1993) and subjects carrying two copies of this gene have a 15-fold likelihood of developing SAD, and at an earlier than those lacking the gene (Altmann et al., 2014; Mahley, 2016).

Both FAD and SAD have abnormalities in the brain circulation (Cortes-Canteli and Iadecola, 2020), together with mitochondrial dysfunction (de la Monte and Tong, 2014; Grimm et al., 2016), which results in the formation of reactive oxygen species causing damage to lipids and proteins (Cai and Tammineni, 2017). Cognitive status was found to be negatively correlated with the level of oxidation of lipids and proteins in the frontal cortex of subjects with SAD (Ansari and Scheff, 2010). This was accompanied by activation of astrocytes and microglia and the release of pro-inflammatory cytokines, particularly TNFα (Fischer and Maier, 2015) and IL-1β (Griffin and Mrak, 2002). The cytokines also produce reactive oxygen and nitrogen species further exacerbating neurodegeneration (Smith et al., 2012). Oxidative stress (Butterfield, 2021) and elevated pro-inflammatory cytokines (Romero-Sevilla et al., 2022) are present in the brains of subjects with MCI.

## Role of activated microglia and astrocytes in neuronal damage

Damage-inducing ligands released from injured cells act on receptors in microglia to produce an intracellular signaling cascade via adaptor proteins, like Myeloid differentiation primary response 88 and Interleukin-1 receptor (IL-1R) associated kinase. These activate a series of mitogen-activated protein kinases (MAPKs), extracellular signal-regulated kinase, c-Jun N-terminal kinase, p38 MAPK and the NOD-, LRR- and pyrin domain-containing protein 3 (NLRP3) inflammasome (Blevins et al., 2022; Holbrook et al., 2021), through dual phosphorylation on Thr and Tyr residues within a conserved Thr-X-Tyr motif (Cargnello and Roux, 2011). Among these kinases, the p38 MAPK pathway is considered as the pivotal regulator of inflammation (Rivera-Cervantes et al., 2015). IL-1β is also released through the activation of NLRP3. It increases nuclear factor kappa-light-chain-enhancer of activated B cells (NF-κB) (Kelly et al., 2003) that promotes cytokine secretion after its translocation to the nucleus (Barnes and Karin, 1997). NF-κB is strongly associated with age in mice and humans (Abbasi et al., 2015) and is also increased in the brains of subjects with neurodegenerative diseases (Granic et al., 2009; Kempuraj et al., 2016).

MAP kinases control the expression and release of inflammatory genes and cytokines in microglia and other immune cells by transcription factors like early growth response protein (EGR1) (Friedle et al., 2011). p38 MAPK activity is elevated in neurons and glial cells in the hippocampus and cortex in patients with AD (Hensley et al., 1999), together with MAPK kinase 6, an upstream activator of p38 MAPK (Zhu et al., 2001). p38 MAPK stimulates pro-apoptotic signaling pathways (Kheiri et al., 2018) and promotes excitotoxicity (Rivera-Cervantes et al., 2015). The expression of p38α MAPK in neurons is associated with the formation of Aβ, inflammation, and tau-induced synaptic dysfunction (Gee et al., 2020).

**Figure 1:**
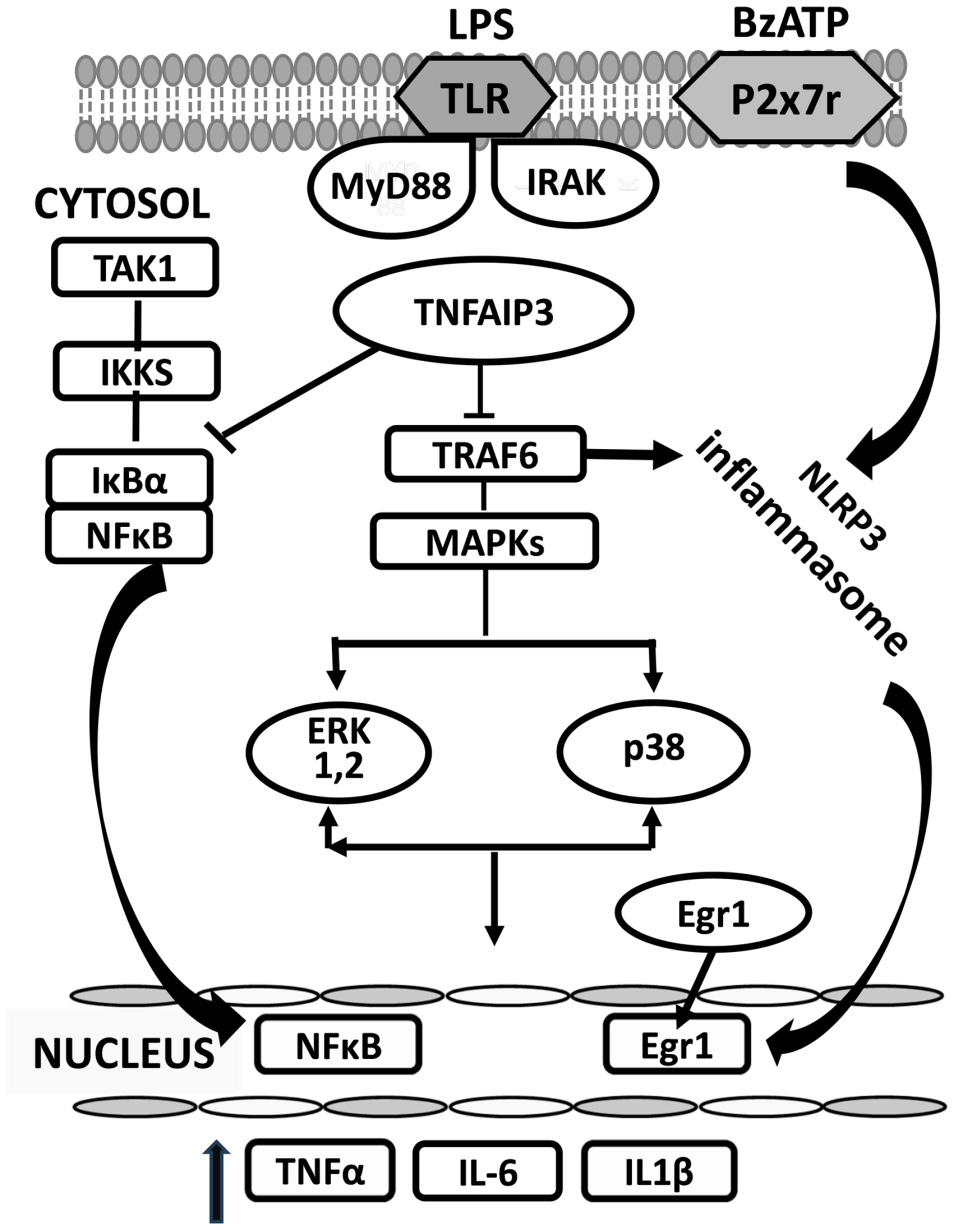
Changes in proteins induced in the cytosol of microglia activated by BzATP and lipopolysaccharide (LPS). LPS (lipopolysaccharide); BzATP (2’-3’-O-(4-benzoyl benzoyl) adenosine 5’-triphosphate); TLR (Toll receptor); MYD88 (myeloid differentiation primary response 88); IRAK (interleukin-1 receptor associated kinase); MAPK (mitogen-activated protein kinase); ERK (extracellular signal-regulated kinase). MyD88 and IRAK are downstream members of the Toll-like receptor (TLR) inflammatory signaling pathway that leads to several functional outputs, including the activation of nuclear factor-kappa B (NFκB), and the release of proinflammatory cytokines. →, activates; ⊥, inhibits.

TNF alpha induced protein 3 (TNFAIP3) is a potent regulator of ubiquitin dependent signals and of immune homeostasis. It is expressed in microglia and other cell types in which it acts as a negative feedback regulator of inflammation (Malynn and Ma, 2019). By controlling ubiquitination of intracellular regulating proteins like Inhibitor of IκB kinase gamma, TNFAIP3 decreases the release of NF-κB from IκBα and its subsequent translocation to the nucleus. It also suppresses proteins like TRAF6, thereby reducing activation both of MAPKs and the NLRP3 inflammasome (Voet et al., 2018) (Figure 1).

## Treatments that reduce the effect of pro-inflammatory cytokines

### TNFα inhibitors

Eternacept, infliximab and adalimumab are three antibodies that bind TNFα and block its actions. They were developed for the treatment of rheumatoid arthritis, ulcerative colitis and Crohn’s disease and can produce a dramatic improvement in these conditions. Although they do not enter the brain, they have been reported to reduce the development of cognitive impairment when given for a period of time to older subjects (Torres-Acosta et al., 2020). This prompted Tobinick et al. (2006) to administer Eternacept by peri spinal injection to a subject with SAD. It produced an immediate and sustained improvement in cognitive function indicating a role for brain TNFα in AD. However, when given to treat peripheral inflammatory conditions TNFα blockers can cause infections, especially reactivation of latent tuberculosis, serious allergic reactions, lymphomas, congestive heart failure and demyelinating disease (Allez et al., 2010; Bongartz et al., 2006). Therefore, there is a need for safer molecules that can prevent oxidative stress and excess cytokine release but do not interfere with their essential activities.

### MAPK p38 inhibitors

An excess release of pro-inflammatory cytokines causing organ damage occurs in several other diseases including, cancer (Denny, 2022), cardiovascular disorders (Papaconstantinou, 2019) and lung disease (Breyer et al., 2012). Recognition of the role of p38 MAPK in propagating inflammation led to synthesis and evaluation of p38 MAPK inhibitors for the treatment of such conditions (Bivona et al., 2023). They were shown to alleviate some of them in experimental disease models (Cohen et al., 2009; Marber et al., 2011). However, when assessed in humans they were withdrawn, either because of lack of sufficient efficacy (Damjanov et al., 2009; Watz et al., 2014), or because of adverse effects, like maculo-papular rash, stomatitis and severe headache (Goldman et al., 2018). Moreover, many of these drugs are unable to enter the brain, or avoid exclusion by p-glycoprotein (Lee and Kim, 2017) which has precluded their use in subjects with AD. Neflamapimod, given at a dose of 40 mg twice daily, lowered cerebrospinal fluid biomarkers of synaptic dysfunction but did not improve episodic memory in patients with mild AD. This trial may have failed because the dose was too low or there were too few subjects in the trial (Prins et al., 2021).

## The peroxisome proliferator-activated receptor *γ* agonists: rosiglitazone

The PPARγ receptor acts as a master regulator of adipocyte differentiation and plays an important role in lipid metabolism and glucose homeostasis. Of the various synthetic ligands of this receptor, rosiglitazone has the highest bioavailability and fewest side effects (Lebovitz, 2002). Rosiglitazone can inhibit the release of proinflammatory cytokines TNFα, IL-6 and IL-1β from lipopolysaccharide-activated RAW264.7 cells (Zhou et al., 2021) and the expression of IL-6 and other inflammatory genes in the cortex of mice after controlled cortical impact (Yi et al., 2008). In diabetic subjects, rosiglitazone (4 mg/day) decreased the levels of NF-κB in the nuclei of circulating mononuclear cells (Mohanty et al., 2004), consistent anti-inflammatory effect. It prevented learning and memory deficits induced in rats by a high fat diet by correcting peripheral insulin resistance (Pathan et al., 2008). Thus, it was considered to have the greatest likelihood of reducing the development of SAD when given to diabetic subjects (Akimoto et al., 2020), because it increases their insulin sensitivity and decrease hepatic triglyceride content (Mayerson et al., 2002). In a phase 2 trial of rosiglitazone in patients with mild-to-moderate AD, a beneficial effect was found on cognition in APOE4-ve individuals (Risner et al., 2006). However, in a larger phase 3 trial of 24 weeks duration no difference was found from placebo in the Alzheimer’s disease assessment scale in those with or without an ApoE4 gene with doses of two and eight mg of Rosiglitazone (Gold et al., 2010). This is not surprising if any potential therapeutic effect on cognitive symptoms results from a reduction in the sequelae of diabetes and not from any direct effect of the drug in the brain. It may have succeeded despite its poor brain penetrability if had it been given at the stage of MCI for long enough to enable its effect on peripheral inflammation and insulin resistance to influence a reduction in memory decline. However, the significant levels of cardiotoxicity reported in subjects receiving the drug could outweigh any potential benefits (Nathan, 2007).

In developing a drug for AD, the major focus should be to prevent the pathogenic processes in the brain leading to cognitive impairment and dementia by giving it at the earliest signs of memory loss, amnestic MCI. Patient identification for initiation of treatment and its successful outcome must depend on clinical and biomarker-based measures that can differentiate those that will develop AD within 2–3 years from those who do not. We highlight our experience with ladostigil as a potential candidate.

## Ladostigil (SPE 100)

Ladostigil, 6-(N- ethyl, N- methyl carbamyloxy)-N propargyl-1(R)-aminoindan hemitartrate is a small molecule that readily penetrates the brain after oral administration and was originally designed to treat subjects with AD. The carbamate moiety of rivastigmine was introduced into rasagiline, a selective monoamine oxidase B (MAO-B) inhibitor to provide AChE inhibitory activity. It was hypothesized that MAO-B inhibition would reduce oxidative stress resulting from the metabolism of biogenic amines and improve apathy and depression, characteristic of subjects with AD (Weinstock et al., 2000). However, ladostigil was found to be a much weaker inhibitor of AChE and MAO-B enzymes *in vitro* than either rivastigmine or rasagiline, respectively (Sterling et al., 2002; Weinstock and Groner, 2008). Nevertheless, chronic administration to rats inhibited both enzymes through the formation of active metabolites. Since the results of a phase 2 trial in subjects with AD that received 80 mg, twice daily did not show any added benefit from MAO-B inhibition over rivastigmine alone further development was discontinued.

At a concentration too low to inhibit AChE or MAO-B, ladostigil decreased the nuclear translocation of NF-κB and phosphorylation of extracellular signal-regulated kinase and p38 MAPK and downregulated gene expression of TNFα, IL-6 and IL-1β in microglia activated by lipopolysaccharide (Panarsky et al., 2012). In SH-SY5Y neuroblastoma cells, ladostigil prevented the fall in the mitochondrial potential resulting from oxidative stress by delaying the opening of voltage-dependent anion channels (Maruyama et al., 2003). Oral administration of ladostigil (1 mg/kg/day) for 6 months to 16-month-old rats that did not inhibit either AChE or MAO-B prevented the decline in memory and increase in activation of astrocytes and microglia in selected regions of the hippocampus and other brain areas involved in the control of memory (Weinstock et al., 2013). Ladostigil also decreased the overexpression of genes encoding pro-inflammatory cytokines, regulating calcium homeostasis, ion channels and those adversely affecting synaptic function, while increasing expression of genes providing neurotrophic support in brain regions associated with learning and memory in the aged rats (Linial et al., 2020). Ladostigil had no effect on the astrocyte and microglial immunoreactivity in the same brain regions in young rats.

**Figure 2:**
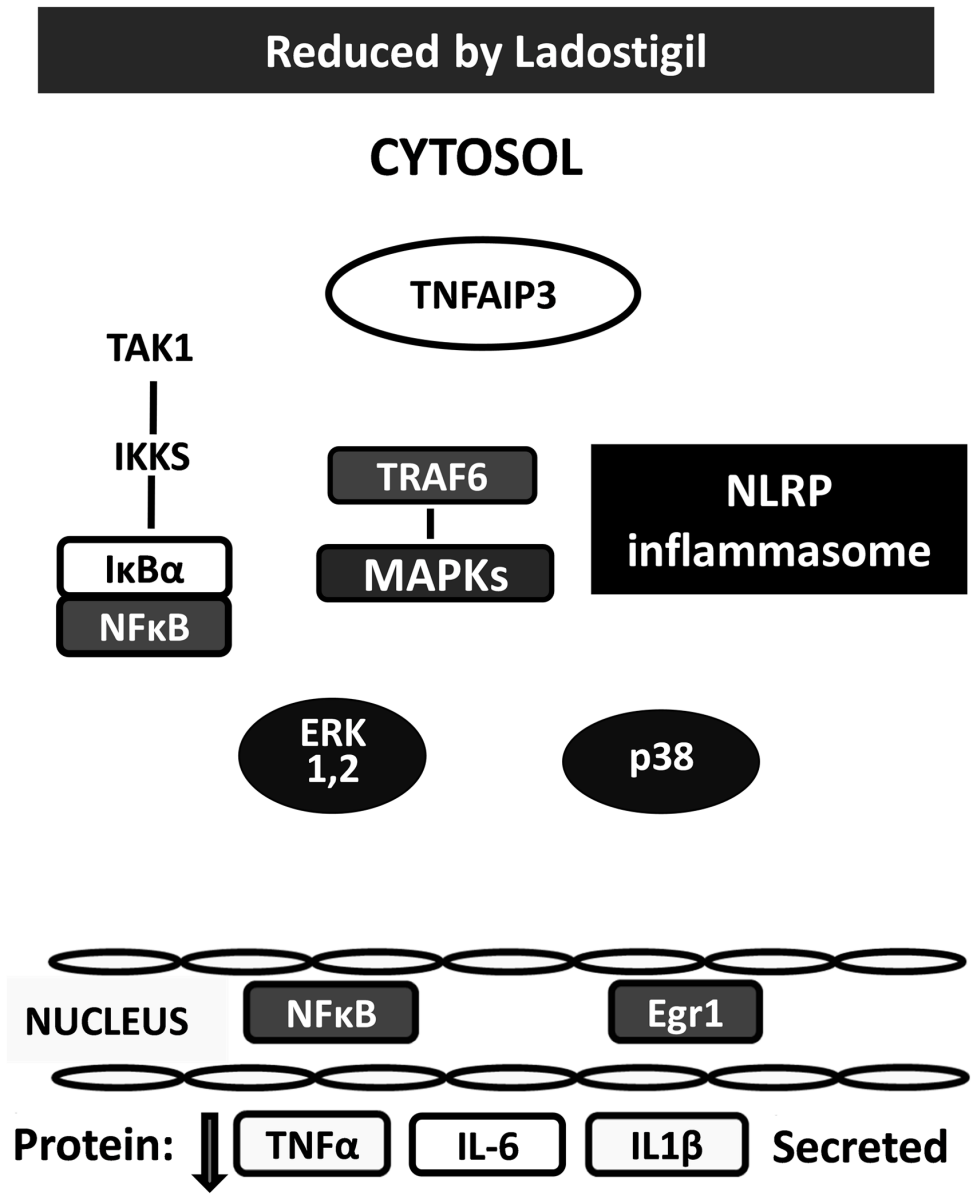
Changes produced by ladostigil in proteins in cytosol and nucleus of microglia activated by BzATP and LPS. Legend as in Figure 1. White colored shapes indicate proteins up-regulated by ladostigil; black colored shapes are proteins downregulated by ladostigil.

In search of the mechanism of action, primary cultures of mouse microglia were activated by a combination of 2′-3′-O-(4-benzoyl benzoyl) adenosine 5′-triphosphate, an agonist of P2x7 receptors and lipopolysaccharide. Ladostigil (0.01–1 nM), concentrations compatible with those found in the cerebral cortex of aging rats in which it prevented development of memory deficits, decreased secretion of IL1β and IL-6 proteins by at least 50% (Reichert et al., 2024). RNA sequential analysis performed on activated microglia showed that ladostigil significantly reduced the transition of EGR1 to the nucleus, while increasing levels of TNFaIP3 in the microglia cytoplasm (Reichert et al., 2024). Restoration to normal by ladostigil of the aberrant signaling of these proteins could explain how it reduced the release of pro-inflammatory cytokines and prevented the morphological and inflammatory changes in brain regions of aging rats (Linial et al., 2020; Shoham et al., 2019) (Figure 2).

**Figure 3:**
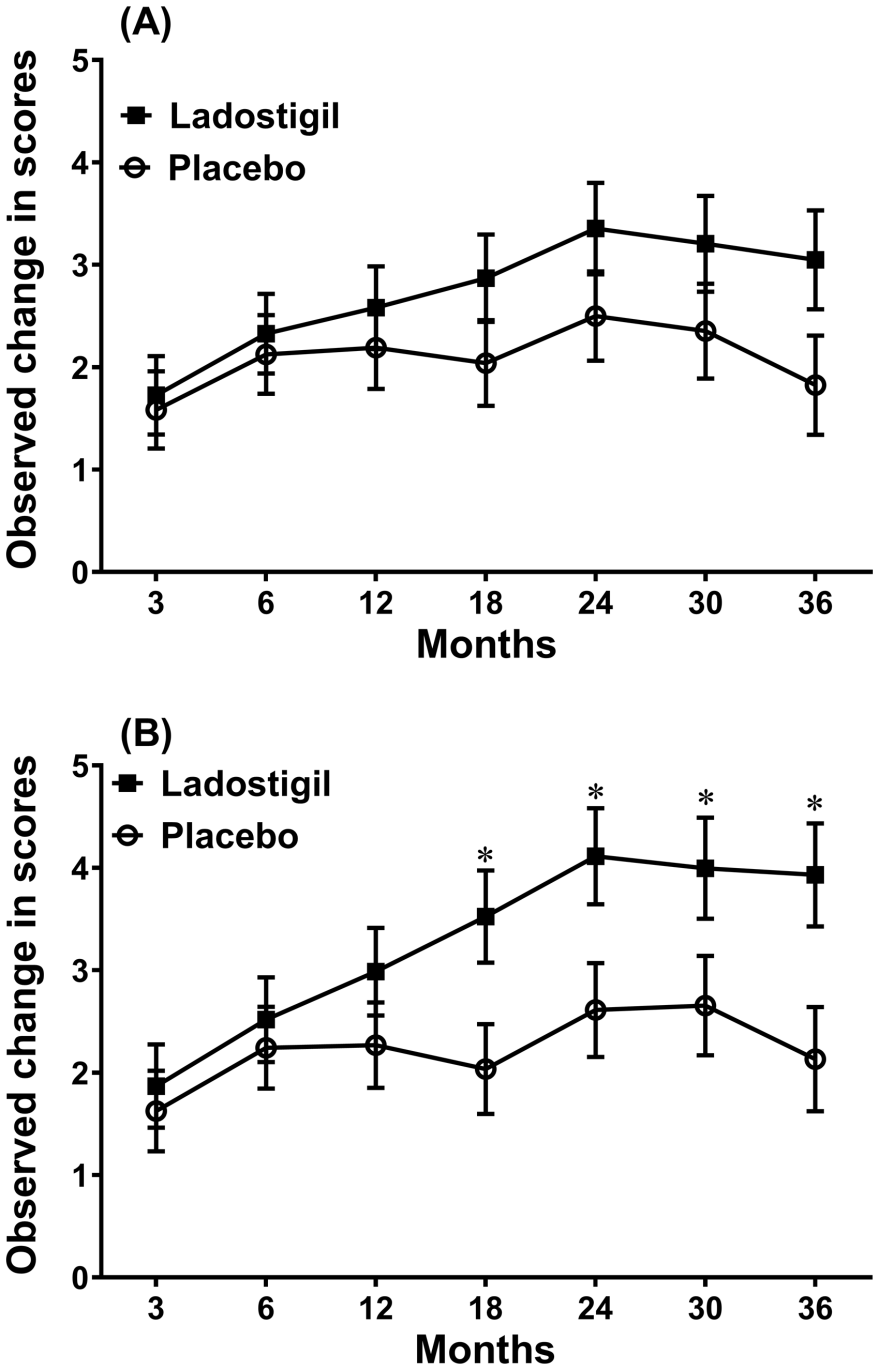
Comparison of the change in score of Rey’s verbal learning delayed recall in subjects that received ladostigil or placebo. **(A)** All subjects. **(B)** Subjects less than 74 years of age. Significant difference between groups * *p* < 0.05. Taken from data in Schneider et al. (2019).

In 2015, a phase 2 trial of ladostigil (10 mg/day) was initiated in subjects with MCI. Unlike the trials with Abs against Aβ, the end point then decreed by the European regulatory authorities was per cent of subjects that converted to AD. Based on previous reports that about 15% of patients would progress to dementia per year (Davatzikos et al., 2011), 100 subjects were included in each drug and placebo group. Since about 30% of the subjects in both groups withdrew from the trial in the course of 3 years and the actual conversion rate in those receiving placebo group was only 7% per year, the study was underpowered because there were also subjects insufficiently impaired to convert within 3 years (Schneider et al., 2019). Moreover, once they converted, they were lost to follow up and all measures. The remaining subjects given ladostigil had a significantly lower mini-mental and higher Schelten’s score (a measure of medial temporal lobe atrophy) than those on placebo. Among subjects without an ApoE4 gene, those in the placebo group progressed at twice the rate of ladostigil-treated group (*p* = 0.028). The age of onset of SAD is younger in ApoE4 carriers (Mahley, 2016). However, nine of them given ladostigil that converted to AD were 6 years older than those receiving placebo and also had a higher CDR-SOB score. When examined in all subjects below 74 years of age, to eliminate this age difference, the rate of decline in the Rey Auditory Verbal Learning Test was significantly lower in those given ladostigil, than in the placebo group (Figure 3).

Unlike monoclonal Abs against Aβ, ladostigil significantly reduced the decline in whole brain and hippocampal volumes. Furthermore, adverse effects were mild and did not differ from those in the placebo group. A new study must consider more recent findings that not all aspects of memory decline are faster in those with MCI than in healthy elderly subjects and use appropriate tests to detect this (Grande et al., 2018). They should also include more accurate measures of magnetic resonance imaging in relevant brain regions and other biomarkers of inflammation and oxidative stress appropriate for MCI subjects.

## Discussion

Alzheimer’s disease is a complex disorder with several risk factors of which the greatest is advancing age. A minority of subjects have genetic abnormalities in the processing of amyloid precursor protein and generation of Aβ, that occur 20 years before appearance of symptoms. The more prevalent SAD is associated with the presence of the ApoE4 gene and with other pathologies including metabolic syndrome, insulin resistance and cardiovascular disease. The recognition that mitochondrial dysfunction and oxidative stress accompanied by excess glial activation correlate better with neuronal damage and memory loss than the presence of Aβ in subjects with SAD has led to the development and evaluation of other medications to address these factors. However, so far, either they were unable to reduce deterioration in cognitive function or their development was discontinued because of serious adverse effects. To be more effective in preventing loss of memory the drugs should be given at the stage of MCI and appropriate tests applied rather than those used in previous studies developed for subjects with AD. Their cost should not prevent them from being readily available to all who need them, and they should be administered orally, reach the brain, and be devoid of adverse effects.
